# CcpA represses the expression of the divergent *cit *operons of *Enterococcus faecalis *through multiple *cre *sites

**DOI:** 10.1186/1471-2180-11-227

**Published:** 2011-10-11

**Authors:** Cristian A Suárez, Víctor S Blancato, Sandrine Poncet, Josef Deutscher, Christian Magni

**Affiliations:** 1 Instituto de Biología Molecular y Celular de Rosario (IBR-CONICET), Departamento de Microbiología, Facultad de Ciencias Bioquímicas y Farmacéuticas, Universidad Nacional de Rosario, Suipacha 531, (S2002LRK) Rosario, Argentina; 2 Microbiologie de l'alimentation au service de la santé humaine (MICALIS), INRA-AgroParisTech, 78850 Thiverval-Grignon, France; 3 INRA-AgroParisTech, 78850 Thiverval-Grignon, France; 4 CNRS, Micalis, F-78350 Jouy-en-Josas, France

## Abstract

**Background:**

In *Enterococcus faecalis *the genes encoding the enzymes involved in citrate metabolism are organized in two divergent operons, *citHO *and *oadHDB-citCDEFX-oadA-citMG *(*citCL *locus). Expression of both operons is specifically activated by adding citrate to the medium. This activation is mediated by binding of the GntR-like transcriptional regulator (CitO) to the *cis*-acting sequences located in the *cit *intergenic region. Early studies indicated that citrate and glucose could not be co-metabolized suggesting some form of catabolite repression, however the molecular mechanism remained unknown.

**Results:**

In this study, we observed that the *citHO *promoter is repressed in the presence of sugars transported by the Phosphoenolpyruvate:carbohydrate Phosphotranserase System (PTS sugars). This result strongly suggested that Carbon Catabolic Repression (CCR) impedes the expression of the activator CitO and the subsequent induction of the *cit *pathway. In fact, we demonstrate that CCR is acting on both promoters. It is partially relieved in a *ccpA*-deficient *E. faecalis *strain indicating that a CcpA-independent mechanism is also involved in regulation of the two operons. Furthermore, sequence analysis of the *citH*/*oadH *intergenic region revealed the presence of three putative catabolite responsive elements (*cre*). We found that they are all active and able to bind the CcpA/P-Ser-HPr complex, which downregulates the expression of the *cit *operons. Systematic mutation of the CcpA/P-Ser-HPr binding sites revealed that *cre1 *and *cre2 *contribute to *citHO *repression, while *cre3 *is involved in CCR of *citCL*

**Conclusion:**

In conclusion, our study establishes that expression of the *cit *operons in *E. faecalis *is controlled by CCR via CcpA-dependent and -independent mechanisms.

## Background

Lactic acid bacteria (LAB) are widely used in food industry due to their capacity to convert sugar into lactic acid. However, they can also metabolize other organic compounds present in the raw material utilized for food fermentation. Citrate metabolism has been extensively studied in LAB from the applied point of view, since this fermentation leads to the production of diacetyl. This compound is the most broadly used butter flavor in dairy industry [1,2] and also contributes to the quality of wine [3].

In LAB, the genes involved in citrate fermentation are usually organized in two operons [4-6]. In these operons, the organization of the genes encoding the holoenzyme of the citrate lyase complex (*citD*, *citE *and *citF*) is extremely well conserved. The clusters also have the accessory genes required for the synthesis and activation of citrate lyase (*citC*, *citG *and *citX*). Two different families of citrate transporters associated to LAB *cit *operons have been characterized [for review see reference [7]]. The 2HCT (2-hydroxycarboxylate) transporter family includes the citrate/lactate exchanger CitP found in *Lactococcus lactis *and *Weissella paramesenteroides *[8], while the proton-coupled citrate-Me^2+ ^symporter of the CitMHS family includes CitH from *Enterococcus faecalis *[9].

We also contributed to the identification of two different oxaloacetate decarboxylases (OAD) linked to the LAB *cit *cluster, i) soluble *citM *[10,11] and ii) the membrane-bound OAD complex (*oadA*, *oadB*, *oadD*), which in *E. faecalis *includes also the novel subunit OadH [6].

Finally, two different transcriptional regulators are involved in the activation of the *cit *operons in LAB: CitI and CitO. CitI belongs to the SorC/DeoR family, and its role in the activation of the *cit *operons was previously established in *W. paramesenteroides *[4,12]. CitI acts in the presence of citrate as an activator, recognizing and binding to two operator sites located in the intergenic region on the *cit *operons [4,12]. CitO, a member of the GntR family, was recently described as the activating factor required for the induction of genes encoding the enzymes involved in citrate metabolism in *E. faecalis*. This activation is mediated by binding of CitO to the *cis*-acting sequences located in the *cit *intergenic region (O1 and O2) in the presence of citrate [6].

Citrate fermentation by *Enterococcus *is relevant, since this group of microorganisms is frequently isolated from the microflora of artisanal cheese [13]. They contribute to cheese ripening and development of their aroma [2]. Early studies [14] showed that *E. faecalis *could co-metabolize lactose and citrate in milk containing yeast extract but could not co-metabolize glucose and citrate in a complex medium. Rea and Cogan analyzed the factors affecting citrate metabolism and found that it was inhibited by the presence of glucose in several *E. faecalis *and *E. faecium *strains [15]. However, the mechanism of glucose-mediated repression of citrate metabolism is poorly understood.

In *Firmicutes*, the global mechanism of CCR is mediated by the pleiotropically acting transcription factor CcpA [for a review see reference [16,17]]. The ability of CcpA to bind its target sites, the catabolite responsive elements (*cre*), is in turn controlled by the presence of its corepressor, serine-phosphorylated HPr (P-Ser-HPr) [18,19]. HPr has been purified from *E. faecalis *[20] and the structures of unphosphorylated [21] and serine-phosphorylated HPr [22] have been determined. Like HPr from other *Firmicutes*, the *E. faecalis *protein can be phosphorylated at histidine-15 using phosphorylated Enzyme I as phosphate-donor and/or at serine-46 by an ATP-dependent HPr kinase, with the former modification slowing the phosphotransfer to sugar-specific Enzyme IIs [23]. The ATP-dependent HPr kinase gene has been cloned from *E. faecalis *[24] and expressed in *Escherichia coli*. The enzyme is bifunctional and acts either as ATP-dependent HPr kinase when bacteria are grown on efficiently used carbon sources or as a P-Ser-HPr dephosphorylating, pyrophosphate-forming phosphorylase when the concentration of ATP and glycolytic intermediates is low. Only P-Ser-HPr, but none of the other HPr forms, is able to form a complex with CcpA active in CCR [19,25].

The results presented in this manuscript suggest a strong repression of the expression of the *cit *operons in *E. faecalis *exerted by CCR. We identified multiple *cre *sites located in the *citH*/*oadH *intergenic region. Furthermore, our results demonstrate that transcriptional repression of the citrate transporter (*citH*) and the transcription factor (*citO*) are caused by the presence of two *cre *sites organized in tandem (*cre1 *and *cre2*), whereas control of the catabolic operon *oadHDB-citCDEFX-oadA-citMG (citCL *locus*) *requires an independent *cre *site (*cre3*). Our studies revealed PTS-mediated CCR mechanisms of the *cit *operons that are partly CcpA-dependent and partly CcpA-independent.

## Results

### Catabolite repression of the *cit *operons occurs in the presence of PTS-sugars

**Figure 1:**
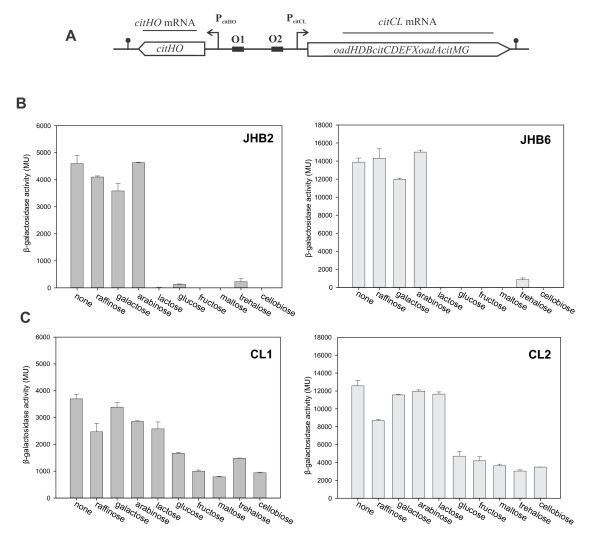
**Effect of different sugars on expression of the *cit *operons**. A) Genetic organization of *E. faecalis cit *metabolic operons. P*citHO*, promoter of the *citHO *operon composed of CitH (Me^2+^-citrate transporter) and CitO (GntR transcriptional regulator); P*citCL *promoter of the *citCL *operon composed of OadHDBA (oxaloacetate decarboxylase membrane complex), CitCDEFXG (citrate lyase and accessory proteins) and CitM (soluble oxaloacetate decarboxylase). O1 and O2 binding sites of the activator CitO. B and C) Influence of diverse PTS and non-PTS sugars on the expression of P*citHO-lacZ *and P*citCL-lacZ *fusions. JHB2 (JH2-2/pTCV-P*citHO*), JHB6 (JH2-2/pTCV-P*citCL*), CL1 (CL14/pTCV-P*citHO*) and CL2 (CL14/pTCV-P*citCL*) were grown in LBC and LBC supplemented with 30 mM initial concentration of different sugars. Levels of accumulated β-galactosidase activity were measured 7 h after inoculation. Error bars represent standard deviation of triplicate measurements.

We recently described that the molecular mechanism underlying activation of the *cit *operons (*citHO *and *citCL*) in *E. faecalis *requires the transcriptional factor CitO [6]. Rea and Cogan had previously suggested that glucose represses citrate metabolism in this bacterium [15]. We therefore studied whether different carbon sources might affect transcription of the genes involved in citrate utilization. To accomplish this task, we measured the activity of the *cit *promoters (P*citHO *and P*citCL*, Figure 1A) by fusing them to the promoterless *lacZ *gene in the vector pTCV-*lac *[26]. β-Galactosidase activity was determined in cell extracts of *E. faecalis *JH2-2 harboring plasmid pTCV-P*citHO *or pTCV-P*citCL*, constructed in a previous work by Blancato *et al*., 2008 (strains JHB2 and JHB6, Table 1) [6].

**Table 1:** *E. faecalis *strains used in this study

Strain	Genotype or description	Source or reference
JH2-2	Cit+	[44,45]
CL14	CcpA deficient	[27]
JHB1	JH2-2 *citO*::pmCitO	[6]
JHB2	JH2-2 (pTCV-P*citHO*)	[6]
JHB6	JH2-2 (pTCV-P*citCL*)	[6]
CL1	CL14 (pTCV-P*citHO*)	This study
CL2	CL14 (pTCV-P*citCL*)	This study
JHB11	JHB1 (pCitO)	[6]
JHB15	JHB1 (pTCV- P*citHO*) (pCitO)	[6]
JHB16	JHB1 (pTCV- P*citCL*) (pCitO)	[6]
JHS1	JHB11 (pTCV-P*citHO-C*_*1*_*C*_*2*_)	This study
JHS2	JHB11 (pTCV-P*citHO-C*_*1*_*C*_*2M*_)	This study
JHS3	JHB11 (pTCV-P*citHO-C*_*2*_*C*_*3*_)	This study
JHS4	JHB11 (pTCV-P*citHO-C*_*2M*_*C*_*3*_)	This study
JHS5	JHB11(pTCV-P*citHO*-*C*_*2M*_*C*_*3M*_)	This study
JHS6	JHB11 (pTCV-P*citCL-C*_*2*_*C*_*3*_)	This study
JHS7	JHB11 (pTCV-P*citCL-C*_*2*_*C*_*3M*_)	This study
JHS8	JHB11(pTCV-P*citCL*-*C*_*2M*_*C*_*3M*_)	This study

First, we studied the effect of the presence of PTS or non-PTS sugars on the expression of both transcriptional fusions in the wild type strain. As shown in Figure 1B, when cells were grown in LB medium containing 1% citrate (LBC) expression of both promoters were active. When non-PTS sugars (raffinose, galactose or arabinose) where added to LBC medium, no repression on the *cit *operons was observed. However, when a PTS sugar was added (glucose, lactose, fructose, maltose, trehalose or cellobiose) to the LBC medium, we found a significant repression of β-galactosidase activity and hence of transcription from both *cit *promoters (93 to 99% of repression) (Figure 1B), which suggests a general CCR mechanism.

### CcpA is controlling *citOH *and *citCL *expression

Because CCR of the *cit *operons was mainly elicited by PTS sugars, it was likely that it followed the general CCR mechanism of *Firmicutes*, which is mediated via the DNA-binding protein CcpA, the corepressor P-Ser-HPr and a *cis*-acting sequence (*cre*). We first tested the involvement of CcpA in repression of transcription from the P*citHO *and P*citCL *enterococcal promoters. To this end, the activity of the *cit *promoters was measured in a CcpA-deficient *E. faecalis *strain (CL14) [27] containing either the pTCV-P*citHO *or the pTCV-P*citCL *plasmid (strains CL1 and CL2, respectively) (Figure 1C). β-Galactosidase activity was determined in cell extracts of *E. faecalis *grown in LBC supplemented with the same PTS and non-PTS sugars, described in Figure 1B. As shown in Figure 1C, no significant repression was observed in the presence of non-PTS sugars and PTS sugars exerted a much weaker repressive effect than in the wild-type strain. However, in these CcpA-defective *E. faecalis *strains the repression was not completely alleviated. A similar observation was reported for other genes controlled by the CCR in *E. faecalis *[27].

**Figure 2:**
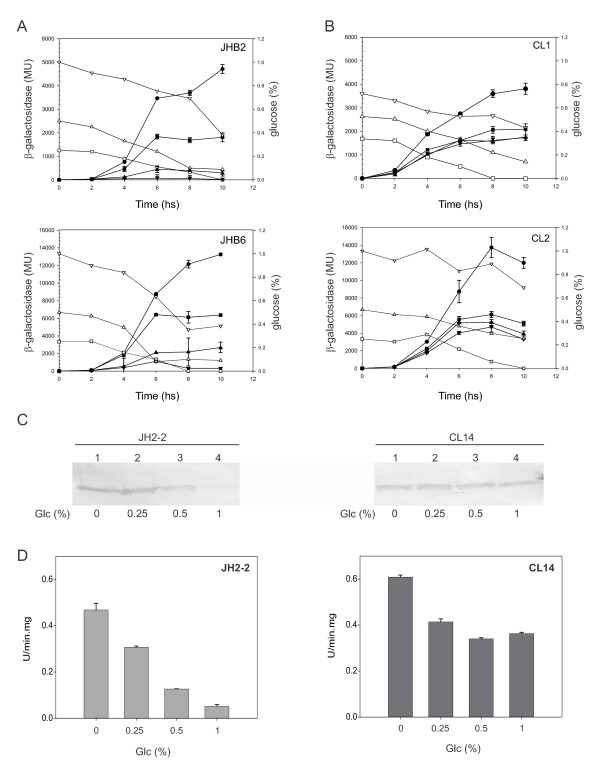
**Effect of glucose concentrations on the expression of *cit *operons, CitO levels and citrate lyase activity**. A and B) JHB2 (JH2-2/pTCV-PcitHO), JHB6 (JH2-2/pTCV-PcitCL), CL1 (CL14/pTCV-PcitHO) and CL2 strains (CL14/pTCV-PcitCL) were grown in LBC (circle) or LBC supplemented with different initial concentrations of glucose: 0.25% (square), 0.5% (up-pointing triangle) and 1% (down-pointing triangle). The corresponding open symbols indicate the remaining glucose concentration in the culture medium (right axis). Levels of accumulated β-galactosidase activity were measured at different times as indicated in the figure. C and D) *E. faecalis *strains were grown in the same conditions of panels A and B, and cells extracts were obtained 7 h after inoculation, C) Western blot analysis was performed with polyclonal antibodies raised against CitO. D) Citrate lyase activity was determined as described previously [5]. Error bars represent standard deviation of triplicate measurements.

Subsequently, we tested whether expression of the *cit *operons depends on the glucose concentration. Hence, we measured the β-galactosidase activity in wild-type and *ccpA *mutant strains carrying either one of the two transcriptional *cit *promoter-*lacZ *fusions. In the wild-type-derived strains (JHB2 and JHB6) β-galactosidase activity decreased when the initial concentration of glucose was raised from 0.25 to 1% (Figure 2A). On the other hand, in the CcpA-deficient strains (CL1 and CL2) activity of the *cit *promoters was independent of the glucose concentration (Figure 2B). These results suggest that the activity of the *cit *promoters is tightly regulated by the availability of glucose and that the pleiotropic transcriptional factor CcpA is involved in this process.

In order to determine whether these differences in transcriptional repression affect the level of the proteins encoded by the *cit *operons, the amounts of CitO and citrate lyase activity were determined. First, a Western blot using antibodies raised against purified CitO was performed with extracts of wild type *E. faecalis *JH2-2 grown during 7 hs in LBC supplemented with different initial concentrations of glucose (0.25%, 0.5% or 1%). A gradual decrease of the intensity of the CitO-specific band accompanied the increase of the glucose concentration (Figure 2C, left panel; lanes 1 to 4). Next, an identical experiment was carried out with the CcpA-deficient strain (CL14) as depicted in Figure 2C (right panel). In this case, CitO levels remained constant despite the increase of the glucose concentration. We also determined P*citCL *repression by measuring the citrate lyase activity in cell extracts. Maximal citrate lyase activity was measured in the wild type JH2-2 strain grown in LB supplemented with 1% citrate (Figure 2D, left panel). However, activity diminished when glucose was added to LBC medium, with maximal repression reached at 1% glucose (90% of repression). Citrate lyase activity was also measured in the CcpA-deficient strain CL14 grown under conditions identical to those used for JH2-2. Only 40% repression was observed in this case, with no significant difference between the activities measured at the different glucose concentrations.

### Both *cit *operons are under the direct control of CCR

**Figure 3:**
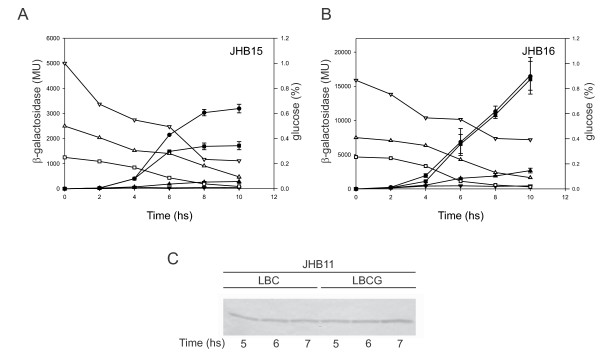
**Effect of different glucose concentrations on the expression of *cit *promoters in a CitO constitutive genetic background**. A and B) JHB15 (JHB11/pTCV-P*citHO*) and JHB16 strains (JHB11/pTCV-P*citCL*) were grown in LBC (circle) or LBC supplemented with different initial concentrations of glucose: 0.25% (square), 0.5% (up-pointing triangle) and 1% (down-pointing triangle). The corresponding open symbols indicate the remaining glucose concentration in the culture medium (right axis). The levels of accumulated β-galactosidase activity were measured at the time points indicated in the figure. Error bars represent the standard deviation of triplicate measurements. **C) **Western blot analysis was performed in the complemented CitO deficient strain (JHB11), that was cultivated for 6 h in LB medium supplemented with citrate 1% (LBC) or citrate 1% plus glucose 1% (LBCG).

The divergent organization of the *cit *genes raises the possibility that the CCR observed could be accomplished by repressing the positive regulator of the pathway (CitO) and the citrate uptake (mediated by CitH). To address this question, CitO was expressed in *trans *autonomously of the P*citHO *promoter (strain JHB11) [6]. In that strain we used the pBM02-derived [28] plasmid, pCitO, in which the expression of *citO *is under the control of the lactococcal P*cit *promoter. As described by Marelli *et al*., 2010 [28], in *E. faecalis *expression of different genes put under control of the P*cit *promoter was constitutive. In the JHB11-derived strains JHB15 and JHB16 (carrying plasmids pTCV-P*citHO *and pTCV-P*citCL*, respectively) the activity of the promoters was determined. From Figure 3A it can be seen that in the JHB15 strain repression occurred over the complete range of glucose concentrations tested, whereas in the JHB16 strain (Figure 3B) repression was only noticeable at higher initial glucose concentrations (0.5% (up-pointing triangle) and 1% (down-pointing triangle)). Western blot analysis indicated that CitO levels remained constant in strain JHB11 independently of whether it was grown in presence of citrate (1%) or citrate (1%) and glucose (1%) (Figure 3C). The results presented in Figure 3 suggest that repression of P*citCL *is directly mediated by CcpA and that repression of P*citHO *is stronger than repression of P*citCL *since P*citHO *but not P*citCL *was repressed at 0.25% initial glucose.

### Multiple *cre *sites mediate the CCR of the *cit *operons

**Figure 4:**
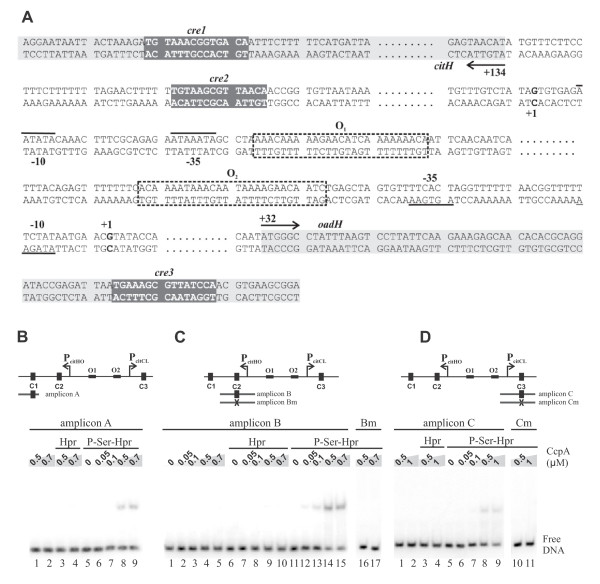
**Binding of CcpA to DNA fragments containing different *cre *sites**. A) Nucleotide sequence of the *citH-oadH *intergenic regions. Locations of transcription start sites are indicated (+1); -10 and -35; regions are shown underlined. Arrows indicate direction of transcription and translation. CitO binding sequences are displayed in dotted boxes and putative *cre *sites in grey boxes. B, C and D) Images of gel shift assays performed with different amplicons (A, B and C respectively) covering each *cre *site or mutated *cre *site amplicons (Bm and Cm), increasing concentrations of CcpA and fixed concentrations of HPr or P-Ser-HPr.

The results presented up to this point show that PTS sugars repress the citrate fermentation pathway through the action of CcpA. A bioinformatic search in the divergent promoter region revealed the presence of three putative catabolite responsive elements (*cre *sites) highly homologous to the *E. faecalis *consensus *cre *site [TG(T/A)NANCGNTN(T/A)CA] [27] and [(T/A)TG(T/A)AA(A/G)CG(C/T)(T/A)(T/A) (T/A)C(T/A)] [29]. *cre1 *(C1) and *cre2 *(C2) are located downstream from P*citHO*; C1 is located in the coding region of *citH *and C2 in the untranslated region at 207-bp and 94-bp, respectively, downstream from the transcriptional start site (TSS) of the *citHO *operon (distances are indicated relative to the center of symmetry). *cre3 *(C3) is located 97-bp downstream from the *citCL *TSS within the coding region of *oadH *(Figure 4).

To address the question whether these putative *cre *sites were recognized by *E. faecalis *CcpA, a His_6_-CcpA fusion protein was overproduced in *E. coli*. The purified fusion protein was used in gel mobility shift assays using DNA fragments corresponding to the individual *cre *sites. The *cre *amplicons were exposed to increasing concentrations of purified CcpA and a fixed concentration of HPr or P-Ser-HPr. FBP was also included in the reaction buffer since its addition enhanced CcpA binding to *cre *sites (not shown). As shown in Figure 4, CcpA without its corepressor did not bind to the *cre *sites under the conditions employed; including HPr in the assay solution did not lead to detectable CcpA-DNA interaction. However, the combination of CcpA with its corepressor P-Ser-HPr resulted in the formation of one retarded complex for each amplicon (Figure 4B, lanes 8 and 9; C, lanes 12-15 and D, lanes 8 and 9). The binding specificity was confirmed by determining that CcpA did not bind to mutated amplicons (Bm and Cm), in which the *cre *site was changed to TaTAcGatTgAAtc (lowercase letters indicate mismatches with the consensus sequence) (Figure 4C, lanes 16 and 17; D, Lanes 10 and 11). A comparison of the binding pattern suggests that the P-Ser-HPr-CcpA complex possesses a 10-fold higher affinity for *cre *site C2 than for C1 or C3, since with 0.05 μM CcpA it is possible to observe the formation of a retarded complex (Figure 4C, lane 12) whereas binding to C1 or C3 required a concentration of 0.5 μM CcpA (lane 8 in Figure 4B and 4D, respectively).

**Figure 5:**
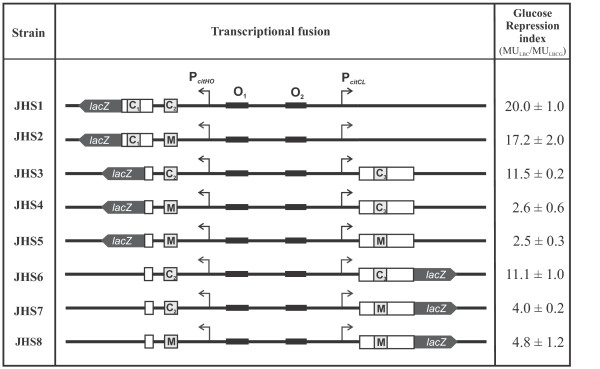
**Schematic representation of the pTCV-*lac *derived plasmids**. Promoter regions of the *citHO *and *citCL *operons are shown. The different *cre *sites are indicated by boxes (C_1_, C_2_, C_3 _and M for mutated *cre *sites). The glucose repression index represents the ratio of accumulated β-galactosidase activity between cell extracts from cultures grown in LBC and LBCG medium (MU_LBC_/MU_LBGC_) for 7 hours.

In order to test the role of these sites in the transcription regulation mechanism mediated by CcpA, a set of DNA fragments corresponding to altered *cit *promoter regions (i.e. *cre *sites deleted or mutated) were fused to the promoterless *lacZ *reporter gene of the pTCV-*lac *vector (Figure 5). Plasmids harboring the P*cit-lacZ *transcriptional fusions were electroporated into the *E. faecalis *JHB11 strain.

We used this strain, in which *citO *is under the control of the constitutive *L. lactis *promoter P*cit*, in order to determine the specific repression mediated by CcpA interacting with the *cre *sites. Accumulated β-galactosidase activity was measured in the JHB11-derived strains grown in the presence of only citrate or of both the inducer citrate and the repressor glucose. In Figure 5, β-galactosidase activities determined 7 hs after inoculation are expressed as glucose repression index (ri = MU_LBC_/MU_LBCG_, where MU_LBC _and MU_LBCG _represent the β-galactosidase activities measured in cells grown in the absence or presence of glucose, respectively). We first studied the effect of alterations in the multiple *cre *sites on expression from the *citHO *promoter. A comparison of the glucose repression index for the transcriptional fusion in strain JHS1, where *cre *sites 1 and 2 are present, with that determined for strain JHS2 containing only functional C1, revealed no significant difference (ri: 20.0 ± 1.0 vs 17.2 ± 2.0) (Figure 5). When C1 was deleted from the *citHO *promoter region we found that C2 was still capable of causing CCR on the *citHO *promoter, but with a slightly lower repression index (ri: 11.5 ± 0.2) (Figure 5, strain JHS3). In contrast, when the C2 site was mutated (strain JHS4) the glucose repression index dropped more than 4-fold compared with strain JHS3 (ri: 2.6 ± 0.6).

We subsequently studied whether the role of C3 in the repression of P*citCL*. The glucose repression index (ri: 11.1 ± 1.0) measured for strain JHS6 indicates that it is submitted to CCR. This repression was diminished in strain JHS7 lacking C3 in the P*citCL *promoter region (Figure 5).

We also found that C3 was not involved in P*citHO *repression, since the presence or absence of C3 had no effect on the repression of this promoter (strains JHS4 and JHS5). Moreover, C2 had no influence on P*citCL *repression because deletion of C2 did not produce a significant difference in the glucose repression index of strains JHS7 (C2 present) and JHS8 (C2 deleted) (Figure 5).

Altogether, these results indicate that *cre1 *and *cre2 *are responsible for CCR of the *citHO *operon, and *cre3 *is the *cis*-acting sequence responsible of the repression of the *citCL *operon.

## Discussion

In this work we demonstrate that citrate metabolism in *E. faecalis *is under the control of the general carbon catabolic repression mechanism and elucidate the details of the CcpA/P-Ser-HPr-dependent molecular mechanism. Clearly, our results establish that CcpA-dependent and -independent mechanisms are involved in CCR of the *cit *operons depending on the repressing sugar employed.

We found that the global transcriptional factor CcpA exerts transcriptional regulation via the three active *cre *sites which allows controlling the expression of the *citHO *operon as well as the catabolic operon *citCL*. Band shift assays showed that the P-Ser-HPr-CcpA complex has a higher affinity for *cre *site C2 than for C1 or C3. Miwa et al. analyzed several *cre *sites from *B. subtilis *and concluded that strong similarity of *cre *sequences to the consensus sequence favors a physiological role and that a more extended palindrome of *cre *sequences correlates with stronger repression [30]. Remarkably, Schumacher *et al*. recently established that P-Ser-HPr-CcpA complex binds to different *cres *with similar affinities. However, it is important to note that this analysis was performed with P-Ser-HPr-CcpA interacting only with *cre *sites belonging to different operators [31]. The difference in affinity that we observed between C1, and C2 or C3 might therefore be related to the surrounding sequences of the *cre *region [32]. This also might explain why C2, although having the highest affinity for CcpA, seems not to be the dominant *cre *in repression.

Interestingly, analysis of the effect of different PTS sugars on the *cit *operons showed significant differences. The presence of lactose in the growth medium produced a strong repressive effect which was completely relieved in the CcpA deficient strain. However, with other PTS sugars, such as glucose, this repressive effect was only partially relieved in the CcpA-defective strain. This result suggests that lactose repression of the *cit *operons is exclusively mediated via CcpA, whereas for the other sugars CcpA-independent mechanisms seem to exist. This observation prompted us to look for alternative PTS repression mechanisms involved in CCR observed in the *cit *operons. First, we searched for phosphorylatable domains in the transcriptional regulator CitO that could regulate its activator function in response to their phosphorylation state [33]. A domain search analysis of the amino acid sequence by means of InterProScan Sequence Search [34] revealed that CitO does not possess neither PRDs nor EIIA or HPr domains. We also dismissed inducer exclusion as possible mechanism of CcpA-independent repression because the *E. faecalis *strain grown in LB in the presence of citrate and glucose maintained the ability to incorporate [^14^C]-citrate (data not shown). Interestingly, Zeng *et al*. suggested that there is a direct involvement of P-Ser-HPr and the glucose/mannose-PTS EIIAB^Man ^(ManL) in CCR of the fructan hydrolase (*fruAB*) and the *levDEFGX *operons [35]. Furthermore, Opsata *et al*. showed that in an *E. faecalis *V583 mutant strain with strong reduction in expression of the mannose PTS operon, the *citE *gene was upregulated 5-fold when compared with the wild type grown in BHI medium (which contains glucose and citrate, among other components) [29]. We constructed a JH2-2-derived mannose PTS deficient strain and a *ccpA *PTS^Man ^double mutant. Unfortunately, we could not find an apparent correlation between the activity of the promoters in the presence of citrate (LBC) and glucose plus citrate. Finally, homologs to CcpN (EF1025) and YqfL (EF2419) were found in the *E. faecalis *genome. These regulators are involved in CcpA-independent CCR in *B. subtilis *[36] and their direct or indirect participation in the regulation of the *cit *operons cannot be ruled out. Recent publications using transcriptome analysis suggested that the *cit *operons might be regulated by Rex (a regulator responding to NAD/NADH ratio) [37] and indirectly by Ers (a PrfA-like regulator) [38]. Nevertheless, their contribution to the regulation in the presence of citrate and PTS sugars remains to be determined. Although convincing evidence for a CcpA-independent mechanism of repression is presented in this work, more experiments will be necessary to elucidate it at the molecular level.

One question which arose from our studies was why does *E. faecalis *regulate citrate transport and metabolism in such a strict way? In *Bacillus subtilis*, citrate uptake interferes directly with the regulation of the Krebs cycle enzymes, which explains why expression of the transporter is tightly controlled [39]. However, citrate transport by enterococcal cells will not cause an imbalance of metabolites of the TCA because *E. faecalis *lacks most of the enzymes of the Krebs cycle. Nevertheless, like *B. subtilis*, *E. faecalis *transports citrate complexed with a well-defined set of bivalent metal ions: Ca^2+^, Sr^2+^, Mn^2+^, Cd^2+^, and Pb^2+ ^[9]. The ability to take up toxic bivalent metal ions in complex with citrate might render *E. faecalis *sensitive to the toxic heavy metal ions in citrate-containing medium. It is possible that the sophisticated regulation of *cit *gene expression allows *E. faecalis *to resist and persist in different environments and to synthesize in controlled form the enzymes necessary for the transport and metabolism of the nutrients in order to optimize its growth.

## Conclusions

In conclusion, this study shows that citrate metabolism in *E. faecalis *is controlled by general Carbon Catabolic Repression. We found that CcpA exerts the transcriptional regulation through three active *cre *sites which allows control of the expression of the *citHO *operon as well as the catabolic operon *citCL*. Thus, this complex regulatory mechanism ensures the control not only of the transcriptional factor *citO *but also of the citrate transporter *citH*, which reduces the uptake of the inducer required by the activator. An extra control point was found in the *citCL *operon which fine-tunes the levels of degradative enzymes encoded by this operon. Also, we found that an independent mechanism of CCR is operative on the citrate operons in this bacterium. All these results contribute to understand how *E. faecalis *controls the hierarchical use of the carbon source that allows it to survive in different habitats and growth conditions.

## Methods

### Bacterial strains and growth conditions

Cultures of *E. faecalis *were grown at 37°C without shaking in 100 ml sealed bottles containing 20-50 ml of Luria-Bertani medium (LB) [40], supplemented with 1% trisodium citrate (LBC) or different carbon sources as indicated with an initial pH of 7.0. The growth medium was supplemented with kanamycin (1000 μg/ml) for strains carrying pTCV-derived plasmids; erythromycin (5 μg/ml) and chloramphenicol (10 μg/ml) for JHB11-derived strains, or erythromycin (150 μg/ml) for the CL14 strain (Table 1).

*E. coli *strain DH5α was used as an intermediate host for cloning and *E. coli *BL21 (DE3) was used for overproduction of His_6_-CcpA. *E. coli *strains were routinely grown aerobically at 37°C in LB and transformed as previously described [40]. Growth was monitored by measuring absorbance at 600 nm in a Beckman DU640 spectrophotometer. Aerobic growth was achieved by gyratory shaking at 250 rpm. Ampicilin (100 μg/ml), erythromycin (150 μg/ml) or kanamycin (50 μg/ml) was included in the medium to select cells harboring ampicillin-, erythromycin- or kanamycin-resistant plasmids. 5-Bromo-4-chloro-3-indolyl-β-D-galactopyranoside (20 μg/ml) (X-GAL) was used to identify recombinant plasmids with DNA insertions that impaired β-galactosidase activity in strain DH5α induced with 0.5 mM IPTG.

### Construction of plasmids with *Pcit-lacZ *transcriptional fusions and β-galactosidase assays

The plasmids bearing the promoter-*lacZ *transcriptional fusions, listed in Table 2, are all derivatives of the pTCV-*lac *vector [26], and the oligonucleotides used in their construction are also indicated in Table 2. In order to mutate the *cre2 *site, the oligonucleotides EfHpromU-Cre2mut_Lo and Cre2mut_Up-EfDpromL (Table 3) were used for the amplification of two overlap extension PCR. These PCR products were used as a DNA template for another PCR using the oligonucleotides EfHpromU and EfDpromL, the amplification products were cloned into the PCR-Blunt II-TOPO vector. The *cre3 *site was mutated by following the same protocol but using the oligonucleotides EfHpromU-Cre3mut_Lo and Cre3mut_Up-EfDpromL (Table 3). Deletion of *cre1 *was carried out by PCR using primers EfbscitN and Efint_Lo. The pTOPO-derived plasmids were digested with *EcoR*I and each released fragment was ligated into the corresponding site of the pTCV-*lac *vector. The desired orientation of the fragments was determined by PCR. Cloned fragments were checked by sequencing at the DNA sequencing Facility of the University of Maine, USA.

**Table 2:** Plasmids used in this study

Plasmid	Characteristics	Oligonucleotides^†^	Reference or source
pGh9	Thermosensitive plasmid carrying erythromycin resistance		[46]
pGEM-T easy			Promega
PCR-Blunt II-TOPO			Invitrogen
pET28a			Novagen
pBM02	pUC18 derivative carrying CRL264 replicon, P*cit *(promoter) and chloramphenicol resistance		[28]
pTCV-lac	Promoterless vector which allows *lacZ *fusion construction		[26]
pmCitO	pGh9 derivative carrying a 500 bp *citO *internal fragment	fcitOU, fcitOL	[6]
pET-CcpA	pET28a derivative expressing His6-CcpA	Ef-ccpAU, Ef-ccpAL	This study
pCitO	pBM02 derivative for expressing CitO in *E. faecalis*		[6]
pTCV-P*citHO*		EfHpromU, EfDpromL	[6]
pTCV-P*citCL*		EfHpromU, EfDpromL	[6]
pTCV-P*citHO-C*_*1*_*C*_*2*_		EfHpromU, EfbsPcitN	This study
pTCV-P*citHO-C*_*1*_*C*_*2M*_		EfHpromU, EfbsPcitN	This study
pTCV-P*citHO-C*_*2*_*C*_*3*_		EfbscitN, Efint_Lo	This study
pTCV-P*citHO-C*_*2M*_*C*_*3*_		EfbscitN, Efint_Lo	This study
pTCV-P*citHO-C*_*2*_*C*_*3M*_		EfbscitN, Efint_Lo	This study
pTCV-P*citCL-C*_*2*_*C*_*3*_		EfbscitN, Efint_Lo	This study
pTCV-P*citCL-C*_*2*_*C*_*3*_		EfbscitN, Efint_Lo	This study
pTCV-P*citCL-C*_*2*_*C*_*3M*_		EfbscitN, Efint_Lo	This study
pTCV-P*citCL-C*_*2M*_*C*_*3*_		EfbscitN, Efint_Lo	This study
		EfbscitN, Efint_Lo	This study

†Oligonucleotide sequences are indicated in Table 3.

**Table 3:** Oligonucleotides used in this study

Oligonucleotides	Sequences (5'-3')
fcitOU	GGAGAATTCAAACGGAACTTAG
fcitOL	TTAACCAAGCTTCTTCTAGGGCAATAC
Ef-ccpAU	GAAGCATATGGAAAAACAAACAATTACC
Ef-ccpAL	GAATGGATCCTTATTTTGTTGAACC
EfHpromU	AGAGGATTCATTACTAAAGATGTAAAC
EfDpromL	CCATCTCGAGTAAATATTCTTTC
EfbsPcitN	ATTGTCTCTCCTTTCACTAATTC
EfbscitN	AAGCTAAAATAGTGAGTAACATG
Efint_Lo	AAACGGAATTCTGGAAACTCTCC
Cre2mut_UP	TACGATTGACACACCGGTGTTAATAAA
Cre2mut_Lo	ACCGGTGTGTCAATCGTATAAAAAAGT
Cre3mut_Up	GAGATTAATAAACGATTGATTCAACGTG
Cre3mut_Lo	CACGTTGAATCAATCGTTTATTAATCTC
EfcitNUp	GGGCCATATGTTACTCACTATTT
Efint4_Lo	TTAGGCTATTTATTCTCTGCGAAAG
EfbsPoadA	GAATTAGTGAAAGGAGAGACAAT
Efbsint_Up	TATCCGCTTCACGTTGGATAAC

Cells were grown overnight in LBC broth and different carbon sources were added to the growth medium at the specified concentrations as indicated in the figures or in the text. Overnight cultures were diluted to an O.D._660 _= 0.08 and grown in LB supplemented with a carbon source until the cells reached early stationary phase. β-Galactosidase activity was measured as described by Israelsen *et al*. [41].

### Protein purification and HPr phosphorylation

The gene encoding the transcriptional regulator CcpA was amplified by PCR using genomic DNA from *E. faecalis *JH2-2 as the template, following a standard protocol. The forward primer Ef-ccpAU introduced a *NdeI *site around the initiation codon of the *ccpA *gene, and the backward primer Ef-ccpAL introduced a *BamHI *site downstream of the stop codon (Table 2 and Table 3). The PCR product was double-digested and ligated into the corresponding restriction sites of vector pET-28a(+) (Novagen). The resulting plasmid, named pET-CcpA, codes for CcpA extended with a 6-histidine tag at the N terminus (Table 2). The correct sequence of the insert was confirmed, and the plasmid was subsequently introduced into *E. coli *BL21 (DE3) for *ccpA *overexpression.

*E. coli *BL21 (DE3) harboring the pET-ccpA plasmid was grown in LB at 37°C until an O.D._600_= 0.6 was reached. Next, CcpA expression was induced by addition of 0.5 mM IPTG. Following an overnight culture, cells were harvested by centrifugation and resuspended in ice-cold Tris-HCl buffer (50 mM, pH 8.0), containing 1 mM phenylmethylsulfonyl fluoride, 1 mM dithiothreitol, 300 mM NaCl and 5% glycerol. Cells were disrupted by passing them through a French Pressure cell. The suspension was centrifuged and the supernatant was mixed with nickel-nitrilotriacetic acid agarose (Novagen). His_6_-CcpA was eluted with imidazole and the purified protein was dialyzed against binding buffer (25 mM Tris-HCl, pH 6.6, 150 mM NaCl and 10% glycerol) and stored at -80 °C for further studies.

*Lactobacillus casei *HprK/P(V267F) and *Enterococcus casseliflavus *HPr were overproduced using pQE30 vector and purified following a standard protocol, as described previously [42].

Seryl-phosphorylated *E. casseliflavus *HPr was prepared as described by Mazé *et al*. [43] using *L. casei *V267F mutant HprK/P, which possesses kinase activity but has almost completely lost the phosphorylase function [42]. About 0.5 mg of HPr was incubated for 30 min at 37°C in 1 ml final volume containing also 10 μg of HprK/P(V267F), 50 mM Tris-HCl (pH 7.4), 5 mM MgCl_2_, 1 mM fructose-1,6-bisphosphate (FBP), and 5 mM ATP. To inactivate HprK/P(V267F), the samples were heated for 5 min at 75°C before they were desalted on PD-10 columns (GE Healthcare Life Sciences) to remove ATP and FBP and lyophilized. HPr and P-Ser-HPr were separated by electrophoresis on nondenaturing 12.5% polyacrylamide gels and visualized by staining with Coomassie blue; usually 99% of the HPr was converted into P-Ser-HPr.

### DNA labeling

The synthetic oligonucleotides EfHpromU, Efint4_Lo, EfbsPoadA were labeled at their 5' ends using [γ-^32^P]ATP (NEN PerkinElmer). The labeled oligonucleotides were purified using a Zeba Desalt Spin Column (Thermo scientific). DNA fragments containing different *cre *sites were amplified by PCR; for the amplicons A, B and C we used the primer pairs EfHpromU-EfcitNUp, EfbscitN-Efint4_Lo and EfbsPoadA-Efbsint_Up, respectively. Amplicon Bm was amplified with the primers EfbscitN-Efint4_Lo using pTCV-P*citHO*-C_1_C_2M _as template and amplicon Cm with the primers EfbsPoadA-Efbsint_Up using pTCV-P*citCL*-C_2_C_3M _as template. The amplicons were purified from a 2% agarose gel prior to their use for binding reactions.

### Gel mobility shift assays

Gel mobility assays were performed as follows. CcpA was incubated with 5 μM HPr or P-Ser-HPr in the reaction mix containing 10 mM Tris-HCl pH 7.5, 1 mM DTT, 1 mM EDTA, 50 mM KCl, 20 mM FBP, 0.05 mg/ml herring DNA and 5% glycerol for 15 min at 37°C subsequently DNA was added to the mixture reaching a final concentration of 0.1 nM. After incubation for another 15 min at 37°C, samples were loaded on a 5% polyacrylamide gel. Gels were dried onto Whatman 3MM paper and exposed to a storage phosphor screen, and band patterns were detected in a GE Healthcare Life Sciences 840 Phosphorimager.

### Citrate lyase activity

To determine citrate lyase activity, cultures of *E. faecalis *JH2-2 and CL14 were grown for 7 hours in LB supplemented with 1% citrate and different glucose concentrations (0.25, 0.5 and 1%). Cells were harvested and resuspended in 200 μl of 100 mM phosphate buffer (pH 7.2) supplemented with 3 mM MgCl_2 _and 1 mM phenylmethylsulfonyl fluoride. Total protein extracts were prepared by treating the cells with 20 U/μl mutanolysin (Sigma) for 20 min at 37°C. Cells were then vortexed with glass beads (425-600 microns, Sigma) and cell debris was removed by centrifugation. The assay mixture contained 100 mM potassium phosphate buffer (pH 7.2), 5 mM trisodium citrate, 3 mM MgCl_2_, 0.25 mM NADH, 25 U of malate dehydrogenase (Sigma), and 20 or 40 μg of total protein from different cell extracts in a final volume of 1 ml. Chemical acetylation of citrate lyase was performed by incubating protein extracts for 5 min at 25°C with 5 mM acetic anhydride and then used immediately for determination of citrate lyase activity. NADH oxidation was measured in a spectrophotometer at 340 nm. One unit of enzyme activity is defined as 1 pmol of citrate converted to acetate and oxaloacetate per min under the conditions used [5].

### Western blot analysis

*E. faecalis *strains JH2-2, JHB11 and CL14 were grown individually at 37°C in LB medium supplemented with 1% citrate and different glucose concentrations (0.25, 0.5 and 1%). Cells were harvested by centrifugation and crude extracts were prepared by vortexing cells with glass beads (425-600 microns, Sigma). Proteins from cell extracts were separated by sodium dodecyl sulfate-polyacrylamide gel electrophoresis (SDS-PAGE) on a 12% polyacrylamide gel and transferred to a nitrocellulose membrane by electroblotting.

Proteins were detected with rabbit polyclonal antisera raised against CitO of *E. faecalis*. Antibodies were visualized by using goat anti-rabbit immunoglobulin G-AP secondary antibodies (Bio-Rad).

### Analytical methods

Glucose concentrations were determined enzymatically with a glucose oxidase-peroxidase based system following the protocol provided by the supplier (Wiener Labs test kit).

## Authors' contributions

CAS carried out the molecular genetic studies, participated in the β-galactosidase activities and protein purification. VSB carried out the molecular genetic studies, participated in the band shift assay and helped to draft the manuscript. SP participated in the purification of the proteins and Band shift assay. JD participated in the coordination and helped to draft the manuscript and CM participated in experiment design, coordination and helped to draft the manuscript. All authors read and approved the final manuscript.
